# Simple Endocardial Running Suture Technique for Concealing Prosthetic Material in Mitral Valve Annulus Cerclage to Prevent Hemolysis

**DOI:** 10.21470/1678-9741-2024-0309

**Published:** 2025-05-20

**Authors:** Paulo José de Freitas Ribeiro, Antonio Carlos Menardi, Fabio Luis-Silva, Andre Luppi, Paulo Roberto B. Evora

**Affiliations:** 1 Department of Surgery and Anatomy, Division of Cardiovascular and Thoracic Surgery, Faculdade de Medicina de Ribeirão Preto, Universidade de São Paulo, Ribeirão Preto, São Paulo, Brazil; 2 Clínica Cirúrgica Coração e Pulmão (CECORP), Ribeirão Preto, São Paulo, Brazil; 3 Department of Clinical Medicine, Faculdade de Medicina do Centro Universitário Barão de Mauá, Ribeirão Preto, São Paulo, Brazil; 4 Cardiovascular Imaging Research Center, Massachusetts General Hospital, Harvard Medical School, Boston, Massachusetts, United States of America

## INTRODUCTION

Hemolytic anemia following mitral valve replacement (MVR) is a rare condition, occurring in < 1% of the cases^[1^-^3]^. The most recognized contributor to this condition involves a regurgitant jet colliding with the valvular apparatus, which, in MVR, includes prosthetic materials such as pericardial strips, prosthetic rings, and protruding suture materials^[2^,^4]^. Early case reports of hemolysis following suture repair of the mitral valve are available in the literature and illustrate this scenario^[5^-^7]^.

**Fig. 1:**
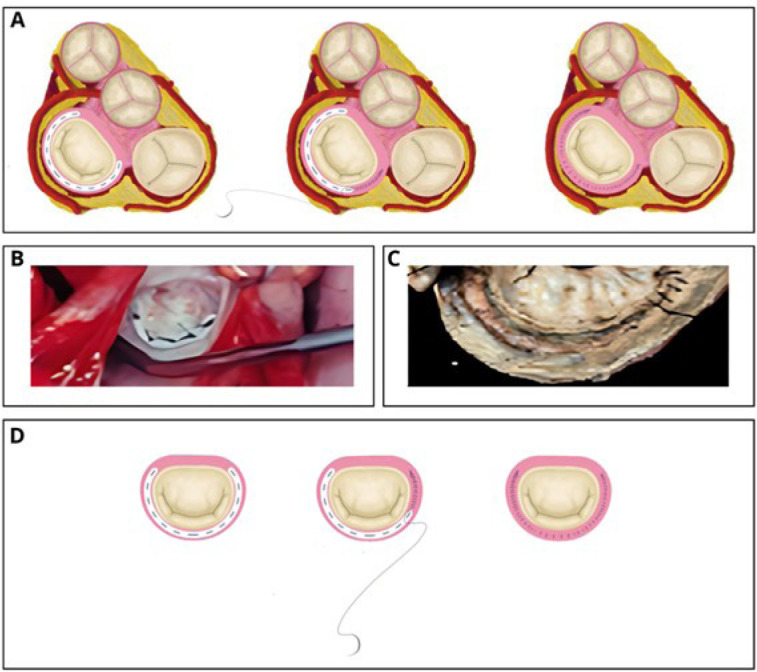
Endocardial running suture technique for concealing prosthetic material in mitral valve plasty. (A) Mitral annuloplasty: partial running suture and completed suture. (B) Intraoperative view of a mitral plasty with a bovine pericardial strip. (C) Necropsy specimen showing non-endothelialized prosthetic material. (D) Schematic views illustrating the endocardial running suture on the mitral valve annulus.

Reintervention, whether by re-repair or valve replacement, must be a safe and effective approach for relieving hemolysis. However, later reports of hemolytic anemia in patients with annuloplasty revealed poor endothelization of the ring, primarily caused by residual regurgitant jets. Early postoperative regurgitation prevents proper endothelization of annuloplasty rings and predisposes to hemolytic anemia^[7^,^8]^ (Figure 1C).

At our institution, hemolysis following MVR occurred in two pediatric patients. Other potential causes were excluded, and the condition was attributed to the prosthetic material used in the annulus. One patient required ferrous sulfate supplements, while the other needed surgical intervention, which involved replacing the Teflon™ felt with a piece of bovine pericardium^[7]^.

Previous studies have discussed strategies to mitigate the adverse effects of prosthetic material in MVR. Salvador et al.^[9]^ introduced the use of an autologous pericardial strip as an alternative to bovine pericardium. The evidence of prosthetic material contributing to hemolysis and our experience with these two cases motivated us to explore new approaches to prevent this condition. We hypothesized that concealing prosthetic material in mitral valvuloplasty could reduce red cell injury, preventing hemolysis. In addition, this technique could also potentially reduce the risk of thrombosis. The aim of this brief communication is to introduce, for the first time, the concept of a suture technique that invaginates prosthetic material, potentially reducing the undesirable effects previously discussed.

### The Technique

After completing the annuloplasty, an invaginating running suture is placed along the entire length of the annuloplasty, plicating the endocardial tissue to cover the prosthetic material (Figure 1).

## COMMENTS

To our knowledge, the literature does not contain any previous presentations of this concept. It is not intended to replace the original MVR technique; it serves to inspire the exploration of alternative approaches for specific scenarios, particularly in patients requiring reoperation because of hemolysis following MVR. We believe it is worthwhile sharing the technical details because the concept is straightforward and safe, does not involve the introduction of additional foreign material, and adds no significant time to the surgery conceptual technique.

We have presented the original concept of a running suture technique designed to conceal prosthetic material and prevent hemolysis.

**Table:** 

Authors’ Roles & Responsibilities	
PJFR	Substantial contributions to the conception or design of the work; or the acquisition, analysis, or interpretation of data for the work; drafting the work or revising it critically for important intellectual content; agreement to be accountable for all aspects of the work in ensuring that questions related to the accuracy or integrity of any part of the work are appropriately investigated and resolved; final approval of the version to be published
ACM	Substantial contributions to the conception or design of the work; or the acquisition, analysis, or interpretation of data for the work; drafting the work or revising it critically for important intellectual content; agreement to be accountable for all aspects of the work in ensuring that questions related to the accuracy or integrity of any part of the work are appropriately investigated and resolved; final approval of the version to be published
FLS	Substantial contributions to the conception or design of the work; or the acquisition, analysis, or interpretation of data for the work; drafting the work or revising it critically for important intellectual content; agreement to be accountable for all aspects of the work in ensuring that questions related to the accuracy or integrity of any part of the work are appropriately investigated and resolved; final approval of the version to be published
AL	Substantial contributions to the conception or design of the work; or the acquisition, analysis, or interpretation of data for the work; drafting the work or revising it critically for important intellectual content; agreement to be accountable for all aspects of the work in ensuring that questions related to the accuracy or integrity of any part of the work are appropriately investigated and resolved; final approval of the version to be published
PRBE	Substantial contributions to the conception or design of the work; or the acquisition, analysis, or interpretation of data for the work; drafting the work or revising it critically for important intellectual content; agreement to be accountable for all aspects of the work in ensuring that questions related to the accuracy or integrity of any part of the work are appropriately investigated and resolved; final approval of the version to be published
